# Assessing the impact of China’s universal two-child policy on infant health: evidence from a quasi-experimental study

**DOI:** 10.1093/eurpub/ckag032

**Published:** 2026-04-29

**Authors:** Di Tang, Zhen li Shan, Wen xian Zhan, Xiang dong Gao, Wei lan Ma, Peter C Coyte

**Affiliations:** College of Public Health, Shanghai University of Medicine & Health Sciences, Shanghai, China; Shanghai First Maternity and Infant Hospital, Tongji University School of Medicine, Shanghai, China; School of Nursing and Health Management, Shanghai University of Medicine & Health Sciences, Shanghai, China; School of Public Administration, East China Normal University, Shanghai, China; School of Global Public Health, New York University, New York, NY, United States; Institute of Health Policy, Management and Evaluation, University of Toronto, Toronto, ON, Canada

## Abstract

China’s “one-child policy” limited many households in China to only one child. This policy had an impact on birth outcomes due to the birth order effects, as firstborn infants typically have lower birth weights. This study aimed to estimate the impact of the “universal two-child policy” on birth weight in China by analyzing individual-level data collected from a major tertiary obstetrics hospital located in Shanghai, the largest metropolitan area in China. Medical records for all births were obtained from a major metropolitan obstetrics hospital between 2013 and 2018. Using difference-in-differences (DID) and quantile DID (QDID) methods while controlling for maternal characteristics and socioeconomic factors, we examined the policy’s impact on birth weight. Analyses included stratification by maternal migrant status, age, and delivery mode. Insurance was found to mediate the treatment effect significantly. Analysis of 133 358 live births showed the policy increased birth weight by 21 g, corresponding to approximately 0.04 standard deviations of birth weight in our sample, with effects varying across maternal age groups and residency status. Insurance coverage mediated 41.3% of the total effect on birth weight. The “universal two-child policy” demonstrated beneficial impact on birth weight in China during the study period, particularly affecting older women, Shanghai residents, and those with natural births.

## Introduction

The one-child policy (1979–2015) fundamentally shaped China’s population dynamics and public health outcomes [1]. The policy influenced various health outcomes, particularly birth order effects, with studies showing firstborn infants often have lower birth weights than subsequent births [2, 3].

The one-child policy was most stringently enforced in metropolitan regions, including Beijing, Shanghai, Tianjin, and Chongqin [4]. Ethnic minorities were generally exempt from these restrictions, creating natural comparison groups for policy analysis [4]. Recognizing mounting challenges from population aging and declining fertility rates, China introduced the universal two-child policy in 2016 [5], which universally permitted all families to have a second child, marking a significant shift from prior, more restrictive measures such as the selective two-child policy of 2013 [1, 5, 6].

From a health economics perspective, birth order effects represent a crucial dimension of human capital formation [2, 7]. Birth weight, a critical indicator of neonatal health and predictor of long-term well-being, has been shown to vary significantly by birth parity [8, 9]. Firstborn children often have lower birth weights due to physiological factors, while higher parity is associated with improved uterine blood flow and better outcomes [2, 8].

The enforcement of the one-child policy likely exacerbated negative birth outcomes by restricting birth parity, as nulliparity has been linked to adverse health effects [2, 3, 8]. These health disparities often persist into mid-childhood, influencing development [7, 10, 11]. Parents typically compensate for these outcomes through increased investment in single-child families [7, 11]. Therefore, it is crucial to evaluate the impact of the universal two-child policy on infant birth outcomes, as easing restrictions could mitigate adverse effects associated with nulliparity [5, 12].

The universal two-child policy may influence infant health through multiple pathways beyond parity effects. First, maternal selection and fertility timing may change as the policy allows families with better socioeconomic status (SES) and maternal health to pursue second births, improving average outcomes among second-born children [13]. Second, household resource allocation may be diverted toward prenatal care and maternal nutrition, as families can plan for a second child without penalties, leading to better fetal development [14, 15]. Third, reduced maternal stress from legitimized pregnancies, coupled with better healthcare access, may improve birth outcomes [16]. Legitimization encourages early prenatal care, insurance enrollment, and hospital delivery, all contributing to improved health outcomes.

While existing research has explored the policy’s broader impacts on fertility rates, population aging, and economic development [1, 17], limited attention has been paid to its effects on infant health outcomes. Given the established relationship between birth parity and infant health, we hypothesize that the universal two-child policy’s relaxation of fertility restrictions would lead to improved birth weights, particularly among second births, with effects varying across different socioeconomic groups and maternal characteristics [2, 18].

This study addresses this research gap by examining the effects of the universal two-child policy on infant birth weight in Shanghai, using individual-level data from a major obstetrics hospital. We employ various econometric techniques, including difference-in-differences (DID) and quantile DID (QDID), to evaluate the impact while accounting for maternal, demographic, and socioeconomic factors [5, 12].

## Methods

### Data source and study population

This study used medical records for all births from a leading obstetrics hospital in Shanghai, China, spanning 1 January 2013 to 31 May 2018. The dataset comprised 133 358 live births, including detailed patient demographics and clinical information from inpatient hospital admissions. This hospital, with over 30 000 annual births, provides comprehensive data.

### Sample selection and eligibility criteria

To ensure robust estimates of policy impacts, we excluded births of third or fourth children (*n* = 1150), cases where mothers appeared more than once in the dataset (*n* = 5092), births to ethnic minorities (*n* = 1690) who were generally exempt from family planning policies, and one extreme outlier in birth weight (33 780 g). After applying these exclusion criteria, the final analytical sample consisted of 125 425 live births, comprising 97 325 firstborn and 28 100 second-born children.

### Variables and measurements

The primary outcome variables were birth weight (BW_i_), measured in grams, and low birth weight (LBW_i_), defined as birth weight ≤ 2500 g [19]. Key independent variables included birth parity (S_i_), a binary variable indicating second child (1) or first child (0), and policy implementation (T_i_), a binary variable indicating births occurring in 2016 and subsequent years (1) or before 2016 (0). Covariates included maternal demographic factors (age, age squared, migrant status, ethnicity, nationality, marital status, employment status) and clinical factors (gravida, gestational age, mode of delivery, postpartum hemorrhage).

### DID approach

We employed the DID approach to evaluate the impact of the “universal two-child policy” on infant birth weight [20], and the estimation is implemented using the diff command in Stata [21]. Under the parallel trends assumption, DID controls for time-invariant unobservable individual and macro-level factors, enabling causal identification [20, 21]. Our empirical model is:


(1) $${BW}_{i}=\alpha_{0}+\alpha_{1}S_{i}+\alpha_{2}T_{i}+\alpha_{3}{(S}_{i}\times T_{i})+X_{i}\alpha_{4}+H_{i}\alpha_{5}+\varepsilon i$$

The DID estimator is captured by the coefficient (α_3_) on the interaction term, which examines whether the policy change led to changes in birth weight for second births following policy implementation.

### Heterogeneous effects analysis and robustness assessment

To examine policy impact heterogeneity, we implemented three approaches: (1) QDID analysis to estimate effects at the 25th, 50th, and 75th percentiles of the birth weight distribution; (2) stratified analyses across maternal age (<35 vs. ≥35 years), migrant status (Shanghai natives vs. migrants), and delivery mode (vaginal vs. cesarean); and (3) mediation analysis examining insurance coverage as a potential mediating pathway.

Robustness checks included falsification tests using ethnic minorities and sensitivity analyses for the 2013–2015 transition period. All analyses were conducted using Stata 16.0, with significance set at *P* < .05.

## Results

### Data description and variable definition

Table 1 displays the descriptive statistics for each of the study variables. Binary variables were used to represent migrant status, occupation, marital status, nationality, ethnicity, and mode of delivery. For instance, if a mother was a native of Shanghai, of Han Chinese ethnicity, employed, married, and underwent a C-section, a value of 1 would be assigned to each of these six binary variables.

**Table 1.** Descriptive statistics

Variable	Definition	Mean	SD
Birth weight		3301	474.5
Birth weight for first borne children (S_i_=0)		3289.60	475.16
Birth weight for second borne children (S_i_=1)		3339.96	469.96
Birth weight before the universal two-child policy (T_i_=0)		3306.31	476.68
Birth weight after the two-child policy (T_i_=1)		3287.84	468.81
Proportion of low birth weight babies	1 = birth weight < 2500; 0 = others	0.05	0.21
Second birth (parity > 1)	1 = birth parity more than one; 0 = first birth	0.22	0.42
Age		30.69	3.9
Age square		956.91	246.41
Ethnicity	1 = Han Chinese ethnicity; 0 = Ethnic minority group	0.99	0.11
Occupation	1 = employ; 0 = no-employ	0.94	0.24
Nationality	1 = China; 0 = others	1	0.04
Marriage status	1 = married; 0 = others	1	0.02
Migrant status	1 = Shanghai natives, 0 = migrant mothers	0.64	0.48
Gravida		1.78	1.05
Mode of delivery	1 = cesarean section; 0 = natural birth	0.41	0.49
Gestation week		39.97	0.67
Postpartum bleeding		308.1	148.3
Number of observations		125 425	

After removing observations of ethnic minorities, births of third or fourth children, duplicate records of mothers, and outlier birth weights, our sample was limited to 125 425 live births.

The sample of mothers had an average age of 30.69 years, with 36% classified as migrants. Almost all were married (99%), and about 94% were employed. The vast majority (99%) identified as Chinese and as Han ethnicity (99%). Approximately 41% selected cesarean section as their delivery mode.

The overall mean birth weight was 3301 g in the complete dataset. For first born children, the birth weight averaged 3289.60 g, while for second born children, it was 3339.96 g. Birth weight averaged 3306.31 g before the introduction of the “universal two-child policy,” and 3287.84 g after its implementation. Total birth weight fell after the introduction of the two-child policy, which may be due to a decline in birth weight across all datasets over the study period, particularly for first births. This apparent decrease in average birth weight after policy implementation requires adjustment for confounding factors in subsequent analyses.

### The results of the DID approach

Table 2 presents the DID estimates of the policy’s impact on birth weight. Column (1) displays the unadjusted estimates, while Column (3) takes into account maternal characteristics and socioeconomic factors. Column (5) reports effects on the probability of LBW.

**Table 2.** DID approach of the influence of the “universal two-child policy” on infant birth weight (models 1–2) and the likelihood of having a low birth weight (model 3)

Variables	Birth weight	*P*-value	Birth weight	*P*-value	Probability of low birth weight (BW ≤ 2500 g)	*P*-value
Birth parity more than one* policy ≥2016 (S_i_=1) & (T_i_=1)	17.535	.011	20.732	<.001	0.994	.023
Birth parity > 1 (S_i_=1)	45.792	<.001	88.582	<.001	0.969	<.001
Policy ≥2016 (T_i_=1)	−24.908	<.001	−22.974	<.001	0.999	.343
Age			−6.251	.036	1.001	.674
Age square			0.091	.055	1.000	.410
Occupation			7.020	.112	0.995	.013
Nationality			24.362	.575	1.007	.746
Migrant status			52.252	.027	0.969	.004
Marriage status			9.404	<.001	0.989	<.001
Gravida			21.289	<.001	0.997	<.001
Mode of delivery			94.149	<.001	0.996	.001
Gestational age			11.940	<.001	0.995	<.001
Postpartum bleeding			0.114	<.001	1.000	<.001
Constant	3296.630	<.001	998.412	<.001	2.615	<.001
Observations	125 425		125 425		125 425	
*R^2^*	0.002		0.405		0.338	

Standardized effect = effect (grams)/SD of birth weight (474.5 g, see Table 1).

*Interaction term between birth parity (Si) and policy implementation (Ti).

The results show that the policy had a modest influence on infant birth weight. Without adjusting for covariates, the policy led to an increase in infant birth weight by 17.535 g (Table 2, Column 1, Row 1, *P* = .011). To express this effect in standardized units, we divided the gram-based effect by the sample standard deviation of birth weight (474.5 g; see Table 1), yielding 17.535 ÷ 474.5 = 0.037 SD units. After controlling for maternal characteristics and socioeconomic factors, the introduction of the policy led to a statistically significant increase in birth weight by 20.732 g (Table 2, Column 3, Row 1, *P* < .001), equivalent to approximately 0.044 SD units (20.732 ÷ 474.5). The policy was also associated with a small but statistically significant reduction in the odds of LBW (odds ratio = 0.994, Table 2, Column 5, Row 1; *P* = .023), corresponding to a 0.6% decrease in the odds of LBW. Several maternal factors showed expected associations with birth weight outcomes.

### The results of the QDID approach

To examine how the policy affects different segments of the birth weight distribution, we used the QDID approach [22], with results presented in Table 3. According to our study, the policy had a favorable and statistically significant influence on the entirety of the birth weight distribution, encompassing the lower, middle, and upper segments.

**Table 3.** QDID approach of the influence of the “universal two-child policy” on infant birth weight

Variables	τ = 25%	*P*-value	τ = 50%	*P*-value	τ = 75%	*P*-value
Birth parity more than one* policy ≥2016 (S_i_=1) & (T_i_=1)	18.358	.004	25.602	<.001	18.025	.020
Birth parity > 1 (S_i_=1)	95.948	<.001	90.913	<.001	81.714	<.001
Policy ≥2016 (T_i_=1)	−16.919	<.001	−27.888	<.001	−32.149	<.001
Age	−6.231	.083	−7.593	.049	−9.478	.028
Age square	0.092	.107	0.114	.063	0.140	.041
Occupation	9.445	.074	12.840	.025	2.321	.719
Nationality	84.702	.097	44.853	.422	−34.053	.586
Migrant status	60.016	.033	38.809	.203	17.746	.606
Marriage status	8.010	.002	7.357	.010	11.201	.001
Gravida	17.777	<.001	21.578	<.001	24.892	<.001
Mode of delivery	61.443	<.001	86.794	<.001	116.267	<.001
Gestational age	11.846	<.001	11.951	<.001	11.860	<.001
Postpartum bleeding	0.097	<.001	0.127	<.001	0.209	<.001
Constant	719.688	<.001	989.719	<.001	1353.539	<.001
Observations	125 425		125 425		125 425	

*Interaction term between birth parity (Si) and policy implementation (Ti).

The effects were statistically significant, with an increase of 18.358 g (Table 3, Column 1, Row 1, *P* = .004) in the lower quantile (25th percentile), 25.602 g (Table 3, Column 3, Row 1, *P* < .001) in the middle quantile (50th percentile), and 18.025 g (Table 3, Column 5, Row 1, *P* = .020) in the upper quantile (75th percentile). These results suggest that the policy had a beneficial impact across the birth weight distribution, with the most pronounced effects observed in the middle segment, benefiting infants at varying levels of birth weight.

### Handling the heterogeneity of the DID estimates

Table 4 shows the DID estimates by maternal age, migrant status, and mode of delivery. Using 35 years as the cutoff between younger and older mothers, our results indicate that the “universal two-child policy” had a notable and favorable effect on birth weight across these subgroups.

**Table 4.** DID approach of the influence of the “universal two-child policy” on infant birth weight by age and by migrant status

Variables	Age < 35	*P*-value	Age ≥ 35	*P*-value	Shanghai natives	*P*-value	Migrants	*P*-value	Nature birth	*P*-value	C-section	*P*-value
Birth parity more than one* policy ≥2016 (S_i_=1) & (T_i_=1)	21.886	.001	33.504	.002	22.462	.001	18.556	.024	27.505	<.001	16.716	.043
Birth parity > 1 (S_i_=1)	87.702	<.001	86.730	<.001	87.190	<.001	91.433	<.001	103.610	<.001	85.334	<.001
Policy ≥2016 (T_i_=1)	−21.263	<.001	−39.612	<.001	−22.120	<.001	−23.889	<.001	−30.918	<.001	−18.558	<.001
Age	—	—	—	—	−14.808	<.001	3.019	.516	1.983	.610	0.137	.978
Age square	−0.175	.094	−0.358	.396	0.212	.001	−0.039	.603	−0.084	.186	0.031	.685
Occupation	6.992	.148	7.722	.480	17.814	.011	−2.528	.661	0.720	.896	10.635	.131
Nationality	−48.565	.360	163.522	.034	33.065	.632	18.442	.743	67.120	.180	−54.991	.484
Migrant status	45.482	.072	75.501	.265	6.561	.855	77.118	.015	33.030	.244	87.026	.029
Marriage status	10.908	<.001	2.628	.654	—	—	—	—	2.563	.332	13.300	<.001
Gravida	23.456	<.001	16.480	<.001	22.665	<.001	19.756	<.001	21.796	<.001	22.285	<.001
Mode of delivery	89.520	<.001	115.342	<.001	100.323	<.001	83.003	<.001	—	—	—	—
Gestational age	11.780	<.001	12.715	<.001	11.738	<.001	12.215	<.001	9.944	<.001	13.955	<.001
Postpartum bleeding	0.131	<.001	0.060	<.001	0.114	<.001	0.113	<.001	0.297	<.001	0.036	<.001
Constant	897.736	<.001	31.612	.960	1213.165	<.001	785.676	<.001	1219.019	<.001	658.033	<.001
Observations	104 570		20 855		79 839		45 586		73 720		51,705	
*R^2^*	0.401		0.425		0.383		0.440		0.341		0.482	

*Interaction term between birth parity (Si) and policy implementation (Ti).

Specifically, the policy increased birth weight by 21.886 g (Table 4, Column 1, Row 1, *P* = .001) among younger mothers (<35 years) and by 33.504 g (Table 4, Column 3, Row 1, *P* = .002) among older mothers (≥35 years) [23]. Regarding migrant status, the policy increased birth weight by 22.462 g (Table 4, Column 5, Row 1, *P* = .001) for Shanghai natives and by 18.556 g (Table 4, Column 4, Row 1, *P* = .024) for migrant mothers. For delivery mode, the results revealed that the policy increased infant birth weight by 27.505 g (Table 4, Column 9, Row 1, *P* < .001) for women who had a natural birth and by 16.716 g (Table 4, Column 11, Row 1, *P* = .043) for women who had a cesarean section.

Figure S1 visually summarizes the heterogeneous DID estimates across subgroups. All point estimates are positive and statistically significant, as evidenced by 95% confidence intervals that do not cross zero. The policy effect was largest among mothers aged 35 years or older (33.5 g, 95% CI: 12.2–54.8), approximately 1.5 times the overall effect. Shanghai natives experienced slightly larger gains than migrants (22.5 g vs. 18.6 g), and vaginal deliveries showed greater benefits than cesarean sections (27.5 g vs. 16.7 g). These patterns suggest that policy benefits were most pronounced among groups with higher SES and lower-risk delivery modes.

### The mediating role of insurance on treatment effects: a comprehensive mechanism analysis

Our mediation analysis examined how treatment effects operate through insurance coverage [24] (Table S1). The total effect of the policy on birth weight was 20.73 g (0.044 SD; *P* < .001). The policy significantly increased insurance coverage (Path a: coefficient = 0.0509, *P* = .007), and insurance positively affected birth weight (Path b: coefficient = 8.120, *P* = .003). Bootstrap analysis revealed an indirect effect of 0.413 (t = 3.30, *P* < .001), indicating that insurance mediates 41.3% of the policy’s effect.

We interpret these findings as suggestive evidence that insurance coverage represents a plausible pathway through which the policy affects birth outcomes, rather than definitive causal mediation. The mediation pathway reflects a correlational relationship; potential alternative explanations include concurrent socioeconomic trends, selection effects, and reverse causality. Establishing true causal mediation would require stronger identification strategies, such as instrumental variables or natural experiments.

### Sensitivity tests

We conducted three sensitivity tests to validate our findings. First, utilizing minority ethnic groups—who were historically exempt from family planning policies—as a control group, we performed falsification tests with and without adjusting for maternal socioeconomic factors (Table S2). The absence of significant effects on both birth weight and the probability of LBW in this group corroborates our main findings. Second, a placebo test with a falsified policy implementation date in 2014 revealed no significant effect (β  =  9.249, *P* = .433), supporting our identification strategy’s validity (Table S3). Third, to mitigate concerns about long-term confounding trends, we restricted our analysis to a narrow time window (2015–2017) around the policy change, yielding results (β  =  12.889, *P* = .114) consistent with our main estimates (Table S3).

## Discussion

### Principal findings and economic implications

Our quasi-experimental analysis provides four key findings regarding the Universal Two-Child Policy’s impact on infant health outcomes. First, the policy led to a statistically significant 20.7 g increase in birth weight, demonstrating tangible health benefits. The effects were observed across the whole birth weight distribution, with the most pronounced improvements in the middle segment. This finding aligns with prior research showing that higher birth parity is associated with better health outcomes at birth [7]. Unlike previous studies [25, 26], however, our analysis quantifies the causal effect of a major demographic policy on infant health.

The observed 20.7-g increase in birth weight—equivalent to 0.044 standard deviation units when standardized by the sample SD of 474.5 g (see Section 3.2)—is modest by clinical standards, as clinically meaningful changes are typically defined as 100–200 g [27]. However, at the population level, even small improvements can yield significant public health benefits. Prenatal nutrition supplementation programs typically achieve birth weight gains of 20–60 g [27–29], and our effect size falls within this range of successful interventions. The 0.6% reduction in LBW may have greater implications for high-risk groups, suggesting complementary interventions are needed for substantial clinical impact.

Second, our heterogeneous effect analysis revealed differential policy impacts across demographic and clinical subgroups. Most notably, the birth weight gains were more pronounced among mothers aged ≥35 years (33.5 g increase), Shanghai natives (22.5 g vs. 18.6 g for migrants), and vaginal deliveries (27.5 g vs. 16.7 g for cesarean sections). These differential impacts underscore how maternal age, residency status, and delivery mode significantly moderate policy benefits [12, 30–33]. For instance, the stronger effects among older mothers likely reflect their typically higher SES, which has been shown to mitigate perinatal risks [9]. This finding aligns with previous literature documenting complex interactions between maternal age, parity, and birth outcomes [30, 34, 35]. Moreover, the effects were most pronounced in the middle of the birth weight distribution, suggesting the policy effectively improved outcomes for average-risk pregnancies while having less impact on extreme cases. This finding underscores the importance of considering equity and efficiency in policy evaluations.

Third, heterogeneous policy effects revealed that healthcare infrastructure and socioeconomic factors moderate policy impacts. The stronger effects among Shanghai natives (22.46 g vs. 18.56 g for migrants) reflect differential access to prenatal care and insurance coverage, contextual to Shanghai’s historically stringent one-child policy enforcement [36]. Delivery mode also emerged as a critical moderator, with greater benefits in vaginal births. This aligns with research showing subsequent pregnancies often require repeat cesarean sections, which are associated with increased maternal morbidity [37]. The limited policy benefits in cesarean deliveries highlight important clinical implications for policy effectiveness [4, 31].

Fourth, our mediation analysis revealed that 41.3% of the policy effect operates through insurance coverage. This finding suggests the policy both directly impacts birth weight and indirectly influences it by encouraging increased insurance uptake. The “universal two-child policy” likely heightened families’ healthcare needs, incentivizing them to secure insurance to mitigate financial risks associated with additional costs. This dual pathway is supported by robust direct (20.32) and total effects (20.73), demonstrating how insurance serves not merely as financial protection but as a driver of improved healthcare utilization. These findings suggest targeted insurance expansions could further enhance policy benefits by facilitating access to quality prenatal care.

Compositional changes in the maternal population. The observed decline in average birth weight after policy implementation (from 3306 g to 3288 g) likely reflects shifts in maternal characteristics. Mothers giving birth after 2016 were, on average, 0.6 years older, more likely to have a second child, and more likely to be migrants (see Table S4: Maternal Characteristics Before and After Policy Implementation). These factors—particularly older mothers and migrants with potentially limited healthcare access—could explain the unadjusted drop in birth weight. However, our DID approach accounts for these compositional changes, thereby isolating the true policy effect. After adjustment, we observe a significant 20.7 g increase in birth weight (*P* < .001), confirming the policy’s positive impact.

### Mechanisms and economic theory

Insurance mediation (41.3%) demonstrates that financial protection reduces economic barriers, enabling better prenatal care. The substantial mediation effect (41.3%, *P* < .001) demonstrates that financial protection reduces economic barriers, enabling better prenatal care and improved delivery outcomes. Second, the heterogeneous effects by SES (e.g. resident status) reveal how resource constraints moderate policy benefits. Migrants, who face systemic barriers to healthcare access, exhibited smaller gains compared to Shanghai natives, consistent with economic theories of healthcare access and inequality. Third, supply-side responses, evidenced by differences in delivery mode, demonstrate how provider behavior adapts to policy-induced changes in demand. For example, natural deliveries showed greater benefits, while repeat cesarean sections, often associated with higher risks, limited policy effectiveness.

### Policy design and implementation

Our findings suggest three critical areas for policy refinement. First, targeted interventions are needed to address the migrant-resident gap in policy benefits. Enhanced insurance portability and reduced administrative barriers could significantly improve healthcare equity. Second, optimizing insurance design to expand coverage and reduce copayments could amplify policy effects. Policymakers should consider subsidizing premiums for low-income families and promoting community-based health insurance schemes. Third, resource allocation should account for population heterogeneity, with additional support directed toward areas serving vulnerable populations.

### Limitations and future research directions

Several limitations should be considered when interpreting our results. First, hospital-level selection bias: our data come from a single tertiary obstetrics hospital in Shanghai, China’s most developed metropolitan area. This hospital serves both higher-SES mothers who preferentially seek tertiary care and high-risk referrals from lower-level facilities. These competing selection forces could bias estimates in either direction: the concentration of higher-SES mothers may overstate effects that require resources to realize, while referral of high-risk pregnancies may dilute average gains.

Second, limited generalizability: Shanghai’s advanced healthcare infrastructure, insurance penetration, and historical policy enforcement differ substantially from rural and less-developed regions. Results in those settings may diverge, requiring caution in extrapolating findings to broader populations.

Third, geographic and socioeconomic heterogeneity: the sample primarily represents urban, higher-SES populations. Multi-center studies across varied regions, healthcare settings (primary/secondary/tertiary), and socioeconomic contexts are essential to establish external validity and identify populations that may require targeted interventions.

Fourth, data limitations prevented examination of alternative mediating pathways. Our analysis focused on insurance coverage, but other potential mediators could not be assessed. Three key factors warrant discussion: (1) Prenatal care utilization—The policy likely increased prenatal visit frequency and care quality, but our data does not capture outpatient visits. (2) Maternal nutrition and health behaviors—Families may improve nutrition and health behaviors with a second pregnancy, but we lack pre-pregnancy BMI and nutritional data. (3) Maternal stress and mental health—Reduced anxiety and fear may improve maternal health, but we did not collect mental health data. Future research should incorporate these variables through cohort studies or record linkages to fully assess policy impacts.

In conclusion, while our findings highlight the positive impact of the Universal Two-Child Policy on infant health in an urban tertiary hospital setting, generalizing these results to rural or less-developed regions requires further validation through multi-center, longitudinal studies.

## Conclusion

This study employs a quasi-experimental design to evaluate the universal two-child policy’s impact on infant health in China, utilizing comprehensive hospital data from Shanghai. Three main findings emerged: (1) the policy led to a significant increase in birth weight (+20.7 g, *P* < .001), with heterogeneous benefits across subgroups; (2) older mothers (≥35 years) and Shanghai residents exhibited greater gains, reflecting socioeconomic gradients in health outcomes; and (3) insurance coverage mediated 41.3% of the policy’s effect, highlighting its pivotal role in improving healthcare access and utilization. The policy implications are evident: (1) targeted interventions are essential to reduce disparities, particularly for migrant populations; (2) enhancing the portability and inclusiveness of health insurance systems could amplify policy benefits; and (3) future policies should account for population heterogeneity to optimize maternal and child health outcomes. These insights provide a robust foundation for refining demographic and health policy in urban China.

## Supplementary Material

ckag032_Supplementary_Data

## Data Availability

Our data will not be shared to protect the participants’ anonymity.

## References

[1] Zeng Y , Hesketh T. The effects of China’s universal two-child policy. Lancet 2016;388:1930–8. 10.1016/S0140-6736(16)31405-2 27751400 PMC5944611

[2] Bohn C , Vogel M, Poulain T et al Birth weight increases with birth order despite decreasing maternal pregnancy weight gain. Acta Paediatr 2021;110:1218–24. 10.1111/apa.15598 32981144

[3] Chen H , Wei T, Wang H et al Association of China’s two-child policy with changes in number of births and birth defects rate, 2008–2017. BMC Public Health 2022;22:434. 10.1186/s12889-022-12839-0 35246096 PMC8895506

[4] Tang D , Laporte A, Gao X et al The effect of the two-child policy on cesarean section in China: identification using difference-in-differences techniques. Midwifery 2022;107:103260. 10.1016/j.midw.2022.103260 35131643

[5] Li H-T , Xue M, Hellerstein S, Cai Y. Association of China’s universal two child policy with changes in births and birth-related health factors: national descriptive comparative study. BMJ 2019;366:l4680. 10.1136/bmj.l4680 31434652 PMC6699592

[6] Liang J , Mu Y, Li X, Tang W. Relaxation of the one-child policy and trends in caesarean section rates and birth outcomes in China between 2012 and 2016: observational study of nearly seven million health facility births. BMJ 2018;360:k817. 10.1136/bmj.k817 29506980 PMC5836714

[7] Pruckner GJ , Schneeweis N, Schober T et al Birth order, parental health investment, and health in childhood. J Health Econ 2021;76:102426. 10.1016/j.jhealeco.2021.102426 33529856

[8] Lin L , Lu C, Chen W et al Parity and the risks of adverse birth outcomes: a retrospective study among Chinese. BMC Pregnancy Childbirth 2021;21:257. 10.1186/s12884-021-03718-4 33771125 PMC8004392

[9] Zhou R , Yu H, Qian N et al Secular trends of low birth weight, preterm birth, and small for gestational age in shanghai from 2004 to 2020: an age-period-cohort analysis. BMC Pregnancy Childbirth 2023;23:540. 10.1186/s12884-023-05799-9 37495942 PMC10373378

[10] Hinkle SN , Albert PS, Mendola P et al The association between parity and birthweight in a longitudinal consecutive pregnancy cohort. Paediatr Perinat Epidemiol 2014;28:106–15. 10.1111/ppe.12099 24320682 PMC3922415

[11] Lehmann JY , Nuevo-Chiquero A, Vidal-Fernandez M. The early origins of birth order differences in children’s outcomes and parental behavior. J Human Resources 2018;53:123–56. 10.3368/jhr.53.1.0816-8177

[12] Geng Y , Zhuo L, Zhang R et al The impact of China’s universal two-child policy on total, preterm, and multiple births: a nationwide interrupted time-series analysis. BMC Public Health 2024;24:236. 10.1186/s12889-023-17620-5 38243163 PMC10799358

[13] Barclay KJ , Kolk M. The long-term cognitive and socioeconomic consequences of birth intervals: a within-family sibling comparison using Swedish register data. Demography 2017;54:459–84. 10.1007/s13524-017-0550-x 28194605 PMC5371623

[14] Grossman M. On the concept of health capital and the demand for health. J Polit Econ 1972;80:223–55. 10.1086/259880

[15] Rosenzweig MR , Schultz TP. Estimating a household production function: heterogeneity, the demand for health inputs, and their effects on birth weight. J Polit Econ 1983;91:723–46. 10.1086/261179

[16] Class QA , Rickert ME, Oberg AS et al Within-family analysis of interpregnancy interval and adverse birth outcomes. Obstet Gynecol 2017;130:1304–11. 10.1097/AOG.0000000000002358 29112654 PMC5783305

[17] Lin Y , Zhang B, Hu M et al The effect of gradually lifting the two-child policy on demographic changes in China. Health Policy Plan 2024;39:363–71. 10.1093/heapol/czae008 38334690 PMC11005836

[18] Deng K , Liang J, Mu Y. Preterm births in China between 2012 and 2018: an observational study of more than 9 million women. Lancet Global Health 2021;9:e1226–e1241. 34416213 10.1016/S2214-109X(21)00298-9 PMC8386289

[19] World Health Organization. International Classification of Diseases for Mortality and Morbidity Statistics (ICD-11): Reference Guide. Geneva: WHO, 2022.

[20] Abadie A. Semiparametric difference-in-differences estimators. Rev Econ Stud 2005;72:1–19. 10.1111/0034-6527.00321

[21] Villa JM. Diff: simplifying the estimation of difference-in-differences treatment effects. Stata J 2016;16:52–71. 10.1177/1536867X1601600108

[22] Callaway B , Li T. Quantile treatment effects in difference-in-differences models with panel data. QE 2019;10:1579–618. 10.3982/QE935

[23] Wang S , Yang L, Shang L et al Changing trends of birth weight with maternal age: a cross-sectional study in Xi’an city of northwestern China. BMC Pregnancy Childbirth 2020;20:744. 10.1186/s12884-020-03445-2 33256654 PMC7708914

[24] Tang D , Gao X, Coyte PC. The effects of compulsory health insurance on birth outcomes: evidence from China’s UEBMI scheme. BMC Health Serv Res 2019;19:779. 10.1186/s12913-019-4657-1 31675975 PMC6824087

[25] Tian M-L , Ma G-J, Du L-Y et al The effect of 2016 Chinese second-child policy and different maternal age on pregnancy outcomes in Hebei province, China. BMC Pregnancy Childbirth 2023;23:267. 10.1186/s12884-023-05552-2 37076792 PMC10114327

[26] Yan J , Wang L, Yang Y et al The trend of caesarean birth rate changes in China after the universal two-child policy era: a population-based study, 2013–2018. BMC Med 2020;18:249. 10.1186/s12916-020-01714-7 32928217 PMC7491061

[27] Ota E , Hori H, Mori R et al Antenatal dietary education and supplementation to increase energy and protein intake. Cochrane Database Syst Rev 2015;2015:CD000032. 10.1002/14651858.CD000032.pub3 26031211 PMC12634316

[28] Lumley J , Chamberlain C, Dowswell T et al Interventions for promoting smoking cessation during pregnancy. Cochrane Database Syst Rev 2009;CD001055. 10.1002/14651858.CD001055.pub3 19588322 PMC4090746

[29] Voerman E , Santos S, Inskip H. Association of gestational weight gain with adverse maternal and infant outcomes. JAMA 2019;321:1702–15. 31063572 10.1001/jama.2019.3820 PMC6506886

[30] Huang Z , Zhang Y, Wang J et al Impact of maternal age on birth weight-related adverse outcomes in newborns: a retrospective study in south-central China. BMC Pregnancy Childbirth 2025;25:92. 10.1186/s12884-025-07199-7 39885447 PMC11780902

[31] Li HT , Hellerstein S, Zhou YB et al Trends in cesarean delivery rates in China, 2008–2018. JAMA 2020;323:89–91. 10.1001/jama.2019.17595 31910272 PMC6990817

[32] Li H , Nawsherwan, Fan C, Yin S et al Changes in adverse pregnancy outcomes in women with advanced maternal age after the enactment of China’s universal two-child policy. Sci Rep 2022;12:5048. 10.1038/s41598-022-08396-6 35322808 PMC8943149

[33] Zhou Y , Yin S, Sheng Q et al Association of maternal age with adverse pregnancy outcomes: a prospective multicenter cohort study in China. J Glob Health 2023;13:04161. 10.7189/jogh.13.04161 38038697 PMC10691438

[34] Zhao Z , Zhu H, Liu H et al The optimal childbearing age and birth spacing in China: a multicenter retrospective cohort study. BMC Public Health 2025;25:2983. 10.1186/s12889-025-24466-6 40885958 PMC12398996

[35] Zhu C , Zhang S, Shen L et al Changes in the characteristics and outcomes of high-risk pregnant women who delivered prior to and after China’s universal two-child policy: a real-world retrospective study, 2010–2021. BMC Public Health 2024;24:336. 10.1186/s12889-024-17810-9 38297279 PMC10829306

[36] Tang D , Gao X, Coyte PC. The relationship between internal migration and the likelihood of high-risk pregnancy: hukou system and high-risk pregnancies in China. BMC Pregnancy Childbirth 2021;21:509. 10.1186/s12884-021-03958-4 34266405 PMC8283949

[37] Kozuki N , Lee ACC, Silveira MF, Sania A, Child Health Epidemiology Reference Group Small-for-Gestational-Age-Preterm Birth Working Group. The associations of parity and maternal age with small-for-gestational-age, preterm, and neonatal and infant mortality: a meta-analysis. BMC Public Health 2013;13:S2. 10.1186/1471-2458-13-S3-S3 PMC3847520 24564800

