# Supplementary material for: Assessing the impact of China’s universal two-child policy on infant health: evidence from a quasi-experimental study
Source: Eur J Public Health. 2026 Apr 29;36(3):ckag032. doi: 10.1093/eurpub/ckag032 (PMC13127144; doi:10.1093/eurpub/ckag032)
Supplement: ckag032_Supplementary_Data [file ckag032_supplementary_data.docx]

**Table S1:** Mediation Analysis of Insurance Coverage

| Path | Estimate (SE) | P-value |
| --- | --- | --- |
| Total effect (c): Treatment × Post → Birth weight (g) | 20.73 (5.32) | P < 0.001 |
| Path a: Treatment × Post → Insurance | 0.0509 (0.0068) | P = 0.007 |
| Path b: Insurance → Birth weight (g) | 8.12 (2.22) | P = 0.003 |
| Indirect effect (a × b) | 0.413 | P < 0.001 |
| Proportion mediated | 41.30% | — |
| Observations (N) | 125,425 |  |

*Note:* All models control for maternal age, age², occupation, marital status, migrant status, gravida, gestational age, delivery mode, and postpartum bleeding. Indirect effect estimated via bootstrap (1,000 replications; t = 3.30).

**Table S2:** Falsification test on a sample of ethnic minorities（N = 1,690）.

| Variables | Birth weight | P-value | Birth weight | P-value | Probability of Low birth weight (BW≤2,500 grams) | P-value |
| --- | --- | --- | --- | --- | --- | --- |
| Birth parity more than one* Policy ≥2016 (S_i_=1) & (T_i_=1) | 64.502 | P = 0.298 | 65.071 | P = 0.294 | 27.454 | P = 0.571 |
| Birth parity > 1 (S_i_=1) | 52.277 | P = 0.136 | 79.854 | P = 0.031 | 134.047 | P < 0.001 |
| Policy ≥2016 (T_i_=1) | -22.174 | P = 0.453 | -29.899 | P = 0.315 | -16.747 | P = 0.471 |
| Age |  |  | 59.955 | P = 0.046 | 54.544 | P = 0.021 |
| Age square |  |  | -1.007 | P = 0.033 | -0.837 | P = 0.026 |
| Occupation |  |  | 94.480 | P = 0.029 | 27.643 | P = 0.416 |
| Nationality |  |  | 43.502 | P = 0.231 | 45.335 | P = 0.113 |
| Migrant status |  |  | -86.924 | P = 0.695 | -108.255 | P = 0.533 |
| Marriage status |  |  | -1.647 | P = 0.948 | -8.142 | P = 0.680 |
| Gravida |  |  |  |  | 10.575 | P = 0.322 |
| Mode of delivery |  |  |  |  | 84.308 | P < 0.001 |
| Gestational age |  |  |  |  | 11.740 | P < 0.001 |
| Postpartum bleeding |  |  |  |  | 0.041 | P = 0.236 |
| Constant | 3,264.982 | P < 0.001 | 2,350.086 | P < 0.001 | 201.335 | P = 0.614 |
| Observations | 1,690 |  | 1,690 |  | 1,690 |  |
| *R^2^* | 0.004 |  | 0.012 |  | 0.398 |  |

**Table S3:** Placebo Tests and Time Window Analysis

|  | (1) Birth weight (placebo 2014) | P-value | (2) Birth weight (2015–2017 window) | P-value |
| --- | --- | --- | --- | --- |
| 1.treated#1.fake_post | 9.249 | P = 0.434 | — | — |
| 1.treated#1.post | — | — | 12.889 | P = 0.114 |
| Observations (N) | 125,425 |  | 79,168 |  |

**Table S4: Maternal Characteristics Before and After Policy Implementation**

| **Maternal Characteristics** | **Pre-Policy (2013-2015)** | **Post-Policy (2016-2018)** | **Change** |
| --- | --- | --- | --- |
| Average Age (mean) | 30.51 years | 31.12 years | +0.61 years |
| Second Birth (%) | 21.13% | 25.46% | +4.33% |
| Shanghai Natives (%) | 66.62% | 56.52% | -10.10% |
| Cesarean Section (%) | 40.75% | 42.36% | +1.61% |
| Sample Size (N) | 88,594 | 36,831 | - |

Note: Pre-policy period includes births from January 2013 to December 2015.

Post-policy period includes births from January 2016 to May 2018.
